# Human Papillomavirus (HPV)-Related Multiphenotypic Sinonasal Carcinoma With Intracranial Extension and Rapid Recurrence: A Case Report

**DOI:** 10.7759/cureus.68824

**Published:** 2024-09-06

**Authors:** Alexis N Reinders, John Gao, Timothy D Smile

**Affiliations:** 1 College of Medicine, University of Illinois College of Medicine Peoria, Peoria, USA; 2 Pathology, Pathology Associates of Central Illinois, Springfield, USA; 3 Radiation Oncology, OSF Saint Francis Medical Center, Peoria, USA

**Keywords:** head and neck, hmsc, hpv-mediated multiphenotypic sinonasal carcinoma, radiation, sinonasal carcinoma

## Abstract

HPV-related multiphenotypic sinonasal carcinoma (HMSC) is a rare malignancy of the nasal cavity or paranasal sinuses that often presents with indolent behavior despite aggressive histologic appearance. Herein, we present an unusual case of a patient with HMSC presenting with rapid local recurrence, highlighting the histopathology and diagnostic and therapeutic strategies surrounding HMSC.

## Introduction

HPV-related multiphenotypic sinonasal carcinoma (HMSC), less commonly known as HPV-related carcinoma with adenoid cystic-like features, is characterized by aggressive histopathologic morphology and high rates of local recurrence despite a relatively indolent clinical course and rare metastatic potential [1,2].

Although it was once considered a subgroup of sinonasal adenoid cystic carcinoma (SNACC) in 2013, HMSC was given the distinction as a separate entity from SNACC [3]. The annual incidence of HMSC is unknown due to the relatively recent discovery and fewer than 100 cases reported in the literature over the past 25 years. HMSC most commonly presents in the fifth decade of life but may present from 20 to 90 years [4]. Commonly reported symptoms include nasal obstruction and epistaxis. In contrast to many sinonasal malignancies, women tend to be affected more than men (1.5:1) [5].

Surgery with or without adjuvant radiation therapy (RT) is the mainstay of treatment, and RT has been shown to increase disease-free survival [1]. However, there is no current standard of care for patients with HMSC. RT with or without concurrent chemotherapy is often employed definitively or when complete surgical resection is not possible. Prognosis tends to be favorable as nodal and distant metastases are exceedingly rare [2].

## Case presentation

**Figure 1:**
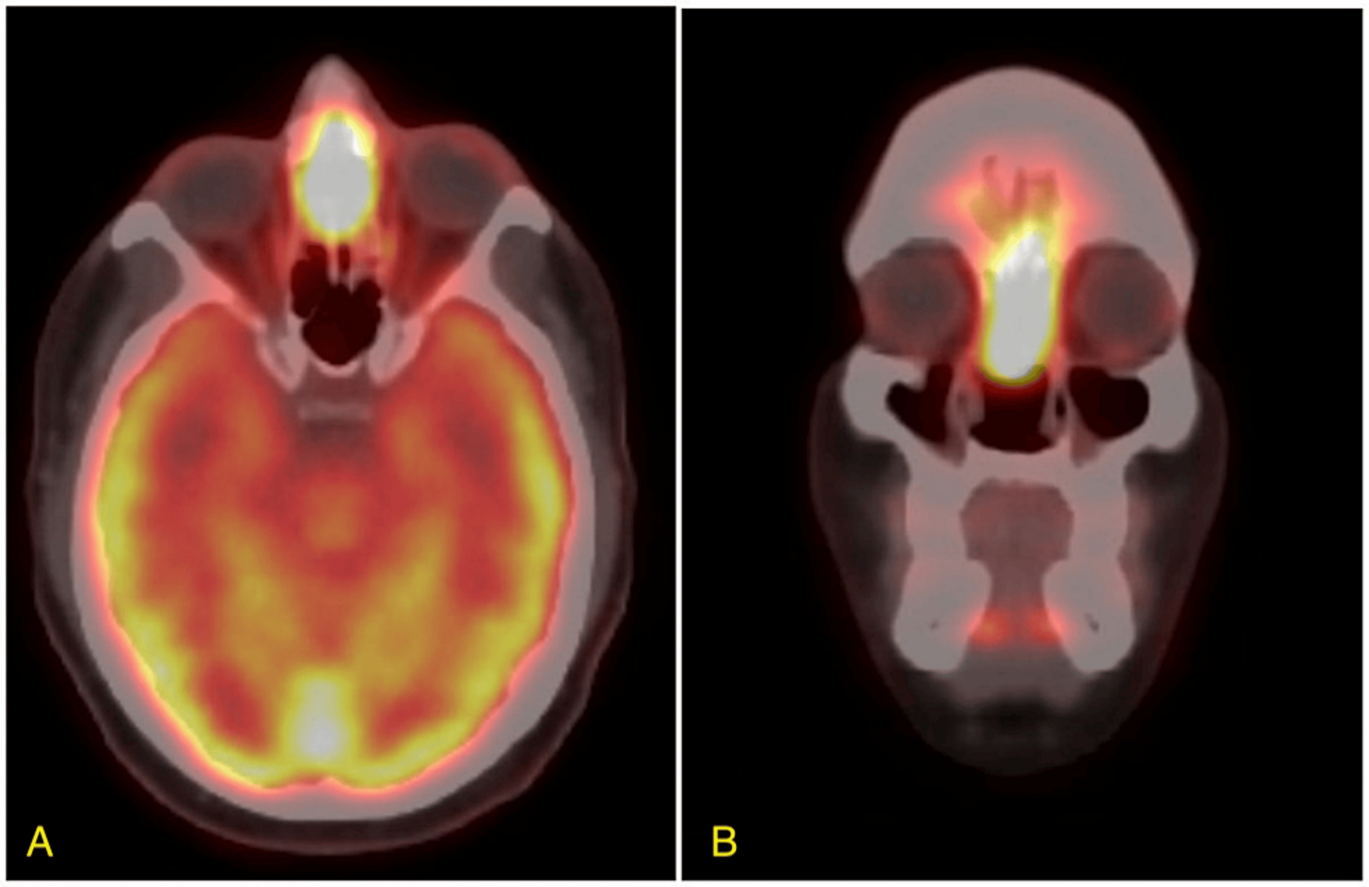
Pre-treatment axial (A) and coronal (B) PET/CT images

**Figure 2:**
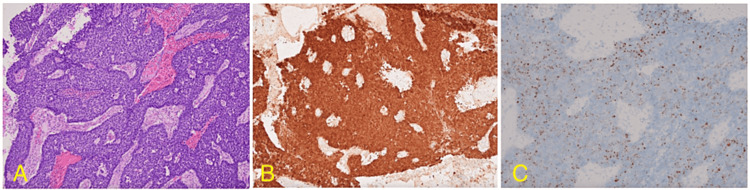
Histopathologic images (A) H&E histology reveals that the tumor exhibits basaloid features with peripheral palisading, a high nuclear to cytoplasmic ratio, and forms solid nests with focal areas of necrosis separated by fibrous bands. (B) Immunohistochemical staining shows diffuse and strong nuclear and cytoplasmic positivity for p16 in the tumor cells. (C) High-risk HPV is detected positively while low-risk HPV is negative based on HPV in situ hybridization. HPV: human papillomavirus

A 49-year-old male initially presented with complaints of nasal congestion and recurrent epistaxis accompanied by mild headaches during the episodes. He was initially discharged with oxymetazoline and was advised to return if symptoms persisted or worsened. Months later, the patient returned with persistent symptoms and was diagnosed with a nasal polyp, prescribed fluticasone and amoxicillin/clavulanic acid, and received a referral to ENT. Transnasal biopsy was initially interpreted as sinonasal intestinal-type adenocarcinoma. CT of the sinuses without contrast showcased a large soft tissue mass measuring 7.1 x 2.5 x 4.2 cm, which occupied the right nasal cavity, causing a mass effect on the septum and medial wall of the right maxillary sinus. Bony infiltration or destructive changes were not observed. A staging PET/CT showed intensely avid fluorodeoxyglucose (FDG) uptake in the 6 x 2 cm primary tumor with erosion and lateral bowing of the medial walls of maxillary sinuses, erosion of the nasal septum and floor of ethmoid sinuses, and a prominent mildly-FDG avid right level 2A lymphadenopathy without evidence of distant metastasis (Figure 1). He underwent fine needle aspiration (FNA) which was negative for carcinoma. Subsequently, the patient underwent endoscopic septectomy and excision of the mass with pathology demonstrating malignancy arising from the right nasal septum pedicle, demonstrating basaloid features with peripheral palisading, a high nuclear to cytoplasmic ratio, and forms of solid nests with focal areas of necrosis separated by fibrous bands (Figure 2). Immunohistochemical staining demonstrated diffuse and strong positivity for p16 in the tumor cells with high-risk HPV detected and low-risk HPV negative on in situ hybridization. The diagnosis was determined to be HMSC.

**Figure 3:**
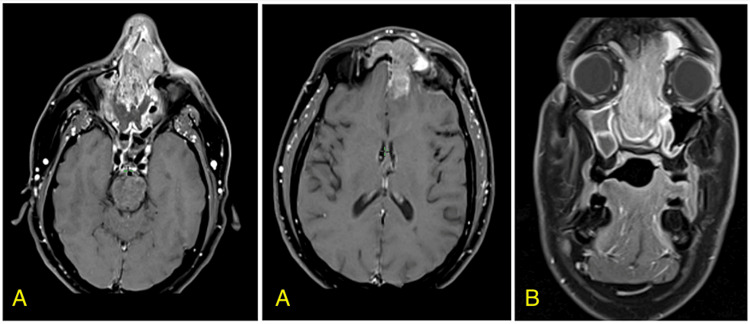
Pre-treatment axial (A) and coronal (B) T1 post-contrast MR images

After surgery, the patient was asymptomatic for approximately two months before developing a left-sided headache that was persistent for six days and not relieved by acetaminophen or nonsteroidal anti-inflammatory agents (NSAIDs). He underwent a follow-up MRI of the orbit, neck, and face which revealed a 2.5 cm lobulated mass within the upper nasal cavity now extending to the left frontal sinus (Figure 3). A subsequent CT of the sinuses without contrast demonstrated a 3.0 x 2.5 x 1.3 cm soft tissue abnormality in the mid-nasal cavity with encroachment on the ethmoid sinus and left frontal recess and there was no evidence of obvious cervical lymphadenopathy. PET/CT demonstrated intense FDG avidity in the nasal cavity lesion with mildly avid uptake of FDG at the level 2A nodes. The patient successfully underwent bilateral sinusotomy, total ethmoidectomy, maxillary sinus mucous membrane removal, and repeat resection of the sinonasal tumor. Margins were negative and surgical pathology confirmed poorly differentiated carcinoma with basaloid features, positive for cam 5.2, p63, and CK5, fluorescence in situ hybridization (FISH) was positive for E6/E7 RNA for which differential diagnosis included HMSC and basaloid squamous cell carcinoma. The case was discussed at a multidisciplinary head and neck (H&N) conference where the recommendation was for adjuvant concurrent chemoradiation (CRT). Two weeks later, the patient was evaluated by radiation oncology where he reported severe pain in the right orbit and frontal sinus areas. The exam revealed nasal bridge edema, tenderness to palpation of the nasal bridge, exophytic intranasal tumor preventing passage of flexible nasolaryngoscope, intact cranial nerves, and no palpable cervical lymphadenopathy. He underwent PET/CT the same day which demonstrated an intensely FDG avid soft tissue mass in the bilateral sinonasal soft tissue with apparent extension above the level of the cribriform plate. MRI of the orbits and H&N with and without contrast was obtained which demonstrated a significantly increased nasal cavity-enhancing mass with intracranial extension into the anteromedial left frontal lobe and into the anteromedial left orbit, but no radiographic lymphadenopathy. Please refer to Figure 1 for pathologic and clinical images.

**Figure 4:**
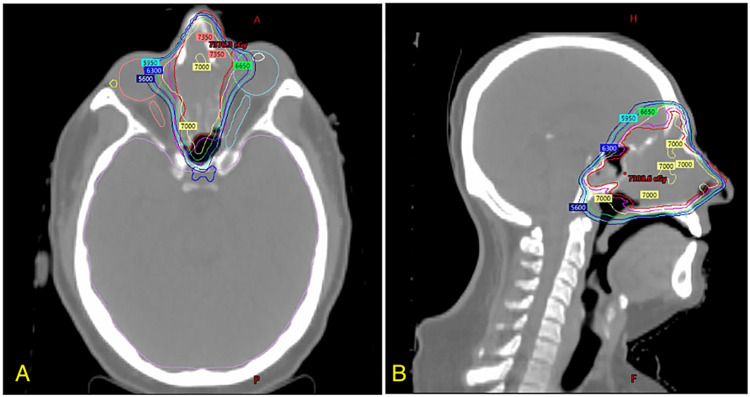
Radiation treatment plan axial (A) and sagittal (B) CT images with radiotherapy isodose lines

The following day, the patient underwent simulation CT with a 5-point aquaplast immobilization mask and was treated with a single fraction of radiotherapy with 4 Gy to the primary tumor using a volumetric modulated arc therapy technique. Two days later, he initiated CRT with 54 Gy/30 fx to the primary disease and bilateral elective cervical nodes and a simultaneously integrated boost to the primary disease to 60 Gy/30 fx. This was followed by a sequential primary tumor boost of an additional 6 Gy/3 fx for a total dose of 70 Gy to the primary tumor (Figure 4). Initially, concurrent bolus cisplatin was given, but this was changed to weekly dosing after the patient developed ototoxicity. The patient experienced acute chemoradiation-related toxicities, including grade 3 opioid-induced constipation requiring brief hospitalization; grade 2 fatigue, mucositis, and dysgeusia; and grade 1 dermatitis, xerostomia, anorexia, epiphora, rhinorrhea, and nausea.

**Figure 5:**
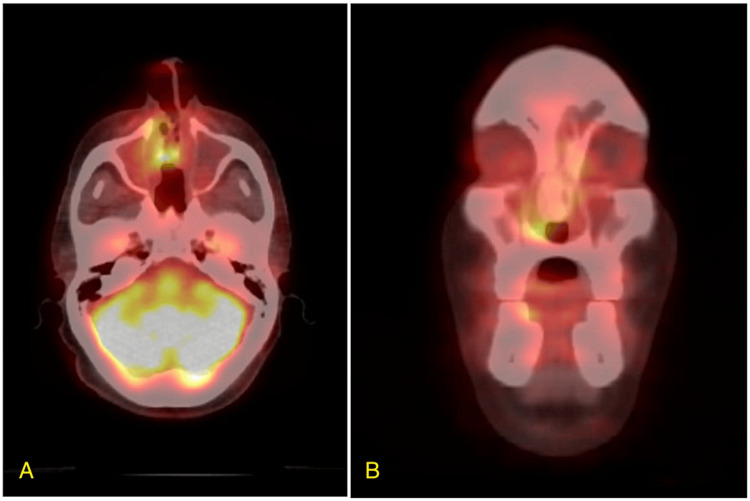
Three-month post-treatment axial (A) and coronal (B) PET/CT images

**Figure 6:**
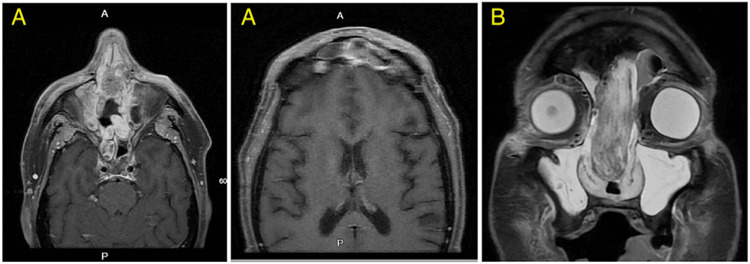
Three-month post-treatment MR images including axial (A) and coronal (B) T1 post-contrast sequences and coronal STIR sequence STIR: short tau inversion recovery

PET/CT three months after completing therapy revealed markedly decreased size and metabolic activity in the treated tumor with some FDG activity in the inferior margin of the treatment site indeterminate for viable disease, but no other suspicious areas of disease were identified (Figure 5). Flexible nasopharyngoscopy was inconclusive due to the mass effect in the nasal cavity continuing to prevent the passage of the fiberoptic endoscope. MRI of the H&N demonstrated a significant reduction in the overall bulk of the tumor with regression in the nasal cavity, frontal and ethmoid sinuses, and cranium (Figure 6). Otherwise, treatment-related and inflammatory changes were noted along with no evidence of pathologic lymph nodes (LNs) in the region. He subsequently underwent salvage resection of the residual mass involving the nasal cavity, ethmoid sinus, and maxillary sinus with pathology revealing inflammatory changes consistent with the treatment effect and showed no evidence of residual viable malignancy. He remains disease-free nine months post-treatment.

## Discussion

HMSC was first identified in 2013 by Bishop et al. as a unique entity among primary HPV-related sinonasal malignancies [3]. Initially, HMSC was considered a subtype of SNACC due to its shared histological features. However, in 2012, Boland et al. highlighted a distinct variant of high-grade SNACC characterized by diffuse p16 positivity and active high-risk HPV [6]. Bishop et al. later distinguished HMSC from SNACC by its specific association with HPV 33, the presence of surface squamous dysplasia, and the absence of MYB gene rearrangement, often found in SNACC [4].

Epidemiology and risk factors

The incidence of HMSC remains unknown due to its rarity and recent discovery. HMSC was identified through retrospective analyses of patients initially presumed to have SNACC [3,6]. The incidence of HMSC is believed to be significantly lower than that of SNACC. SNACC accounts for only 1% of all H&N malignancies and reported cases of HMSC since 2000 number less than 65 [1,7].

HMSC typically presents in the fifth decade of life with a mean age of 54 years, though cases span a wide range of ages [3,4]. Incidence appears to be slightly more prevalent in women than men with a ratio of 1.5 to 1, and no predilection for race has been observed [3-5].

Interestingly, exposure to aerosolized high-risk HPV may be a risk factor for developing HMSC. A case series that recorded occupational data revealed that three out of four patients were nurses or nursing assistants [8]. However, further studies investigating the occupational status of HMSC patients are warranted to confirm or refute this association.

Histopathology

Although the combination of multiphenotypic morphology with HPV positivity in HMSC is fairly specific, this entity can still be confused with other malignancies such as SNACC and nonkeratinizing squamous cell carcinoma (NKSCC) [3-5]. Histologically, all cases of reported HMSC have demonstrated diffusely positive p16 staining, and most commonly have expressed HPV 33 DNA. However, other high-risk HPV DNA expression has been reported, including HPV 35, HPV 16, HPV 56, and HPV 82 [1,8]. HMSC also consistently stains positive for cytokeratin and SRY-related HMG-box 10 (SOX10) [2]. Although usually indolent clinically, HMSC is paradoxically characterized by high-grade histologic features, high mitotic rates, and cell necrosis, and sometimes also characterized by findings of mixed salivary gland and squamous cell differentiation [1,2].

HMSC and SNACC demonstrate positive staining for p16, although the positivity shown in HMSC is strong and diffuse, unlike in SNACC. Both entities contain basaloid and ductal cells, which stain positive for cytokeratin, express CD117, and are reactive to SOX10. Additionally, the basaloid cells often express S100 and p63. As mentioned previously, HMSC can be differentiated from SNACC by the expression of HPV 33, the presence of surface squamous dysplasia, and the absence of MYB gene rearrangement. The MYB/MYBL1 gene fusion occurs in up to 60-70% of SNACC malignancies but has not been reported in HMSC [5].

Another histopathological entity from which HMSC must be differentiated is NKSCC [5]. Similarities between the two include the presence of squamous cell differentiation, and both may have solid morphologic features. HMSC can be differentiated from NKSCC by the presence of both basaloid and ductal cells, as NKSCC characteristically has a smooth stromal interface. Additionally, NKSCC often demonstrates peripheral palisading and is less likely to be positive for HPV, as only 22-50% demonstrate positivity [2].

Staging per American Joint Committee on Cancer eighth edition (AJCC 8th)

According to the AJCC 8th staging system, tumors of the nasal cavity and paranasal sinuses differ in staging according to the primary site. These sites include the maxillary sinus, nasal cavity, or ethmoid sinus. Characteristics of the primary tumor (T), regional lymph nodes (N), and distant metastasis (M) are integrated into a single stage. The degree of invasion determines the T category with T1 tumors limited to one nasal cavity subsite with or without bony invasion, T2 tumors invading two subsites in a single region or extending to involve adjacent region without the nasoethmoidal complex with or without bony invasion, T3 tumors invading the medial wall or floor of the orbit, maxillary sinus, palate, or cribriform plate; T4a tumors invade any of the following: anterior orbital contents, the skin of nose or cheek, minimal extension to the anterior cranial fossa, pterygoid plates, sphenoid or frontal sinuses; and T4b tumors invade either the orbital apex, dura, brain, middle cranial fossa, cranial nerves other than V2, nasopharynx, or clivus. Like other H&N cancers, the nodal staging classification is based on number, size, laterality, and presence of extranodal extension (ENE), with N1 including single ipsilateral LNs 3 cm or smaller in size without ENE, N2a including a single ipsilateral LN 3.1 to 6 cm diameter without ENE, N2b including multiple ipsilateral LNs 6 cm or smaller without ENE, N2c including bilateral or contralateral LNs 6 cm or smaller without ENE, N3a including a LN metastasis greater than 6 cm diameter without ENE, and N3b including any LN metastasis with clinically overt ENE. The M classification is based on the presence or absence of distant metastatic lesions. Prognostic group stages for nasal cavity and ethmoid tumors are generally subdivided into stage IVA (T4aN0-2 or T1-3N2), stage IVB (T4b with any N or any T with N3), and stage IVC (any T or N with M1).

Based on the AJCC 8th edition staging, HMSC typically presents at a low T-stage. A retrospective study encompassing stage data from 39 HMSC patients revealed that the majority (n=23) were classified as T1 (n=16) or T2 (n=7) stages upon initial presentation. Additionally, none of the patients presented initially with nodal or distant metastases [4].

Local recurrence, nodal disease, and metastatic potential

Recent studies have highlighted important distinctions between HMSC and other sinonasal malignancies such as sinonasal undifferentiated carcinoma and SNACC. HMSC has a more favorable prognosis with lower rates of bone invasion and distant metastasis [5]. However, patients with HMSC commonly experience local recurrence as observed in a retrospective study investigating all reported cases (n=57) from 2000 to 2018 [1]. Of the 42 cases with staging information, 25 (60%) presented as early-stage disease (T1/T2). Among the 44 cases with post-treatment follow-up data, 16 cases (36.4%) developed local recurrence. Although extremely rare in the literature, some studies have demonstrated HMSC’s ability to metastasize to distant sites including lung and upper extremity digits [4]. In this study, there were no reports of regional LN metastases or tumor-related deaths [4]. Moreover, Ward et al. postulate that HMSC presenting with bone or perineural invasion would benefit from more aggressive treatment regimens since those features increase the incidence of local recurrence [1].

In more contemporary literature, LN metastasis has been reported. A study conducted between 2017 and 2022 examined the expression of various biomarkers among 40 HMSC patients who underwent surgical resection to investigate the association with survival rates [9]. Among those evaluated, 31 presented with stage I tumors, while those remaining had stage II tumors. Notably, 35% of patients (n=14) were found to have LN metastasis. The study revealed a significant association between LN involvement and the expression of vascular endothelial growth factor (VEGF), human telomerase reverse transcriptase (hTERT), and ProExTMC, suggesting these biomarkers could serve as indicators of LN metastasis risk for future patients. Additionally, high expression of ProExTMC, EGFR, and BAX was associated with poor survival, and a poorer prognosis, suggesting their potential as biomarkers for targeted therapeutics and potential treatment intensification.

A recent case report involving HMSC in a 65-year-old man underscores the significance of conducting a comprehensive assessment for metastasis despite its rarity [10]. The patient initially presented with nasal congestion and epistaxis, leading to the discovery of a mass in his left nasal cavity. Surgery followed by adjuvant proton therapy (66.6 Gy) was initially successful, but two years later the patient developed pulmonary metastasis. Despite chemotherapy and immunotherapy, the disease progressed. This case emphasizes the importance of understanding the behavior of this tumor for proper treatment stratification, particularly regarding adjuvant therapy recommendations. The unique association of HPV subtype 16 with this aggressive clinical course suggests a need for deeper investigation into the relationship between HPV subtypes and clinical behavior in HMSC.

Important distinctions between HMSC and other sinonasal malignancies include the prevalence of LN and distant metastases, time to local recurrence, and overall survival. These differences highlight the critical importance of histopathologic distinction from other sinonasal cancers with very different patterns of failure, treatment strategies, and prognosis. In cases where HMSC is suspected, if a node is positive, distant metastasis is present, or rapid recurrence occurs, additional histopathologic and immunohistochemical studies could be considered to potentially identify another more aggressive diagnosis.

Treatment

Currently, there is no established standard treatment protocol for patients diagnosed with HMSC. However, the primary approach involves surgery, either alone or in combination with adjuvant RT. Aiming for complete eradication with surgical removal of the tumor is crucial. Adjuvant RT is often advised, particularly in cases with positive margins or aggressive characteristics like perineural or bone invasion [1].

In Bishop et al.'s review of 49 cases of HMSC, the treatment modalities varied, with some patients undergoing surgery alone, while others received surgery followed by RT, chemoradiation, or RT alone. Local recurrences were observed in 36% of patients and occurred between 23 and 130 months post-treatment. Treatments for recurrences included surgery, radiation, and chemoradiation. Distant metastases occurred in 5% of patients and were managed with surgical excision and chemotherapy [4].

Despite treatment efforts, HMSC exhibits a notable tendency for local recurrence, as mentioned previously. For patients experiencing local recurrence, the addition of adjuvant RT appears beneficial as it extends disease-free survival. Notably, this combination demonstrates a median disease-free interval of 42 months, compared to 24 months with surgery alone, a difference that approaches statistical significance [1]. Histologic markers such as perineural and bone invasion indicate a more aggressive disease course. Given the potential for late tumor recurrence, prolonged and possibly lifelong surveillance is imperative for individuals diagnosed with HMSC [1].

## Conclusions

In summary, while HMSC generally presents a more favorable prognosis compared to other sinonasal malignancies, its propensity for recurrence and potential for aggressive behavior warrant careful consideration in personalizing the intensity of therapy and follow-up strategies. Further research is needed to identify more specific pathologic markers, to stratify tumors at high risk of rapid recurrence, and to guide optimal therapeutic regimens.
